# Perspectives on equal health and well-being

**DOI:** 10.1080/17482631.2019.1679589

**Published:** 2019-11-12

**Authors:** Staffan Karlsson, Kristina Ziegert, Lars Kristén

**Affiliations:** School of Health and Welfare, Halmstad University, Halmstad, Sweden

As guest editors of the thematic cluster Perspectives on Equal Health and Well-being in the *International Journal of Qualitative Studies on Health and Well-being*, we would like to introduce the theme connected to the five included articles. The articles in this cluster are related to the context of the Swedish healthcare system, which is a socially responsible system with a public commitment to ensure the best health of all citizens. The Swedish healthcare system is based on the belief that all people have equal entitlement to dignity and should have the same rights, regardless of their status in the community, and that those in greatest need should be prioritized. In addition, there should be a reasonable relationship between the costs and the benefits in terms of improved health and well-being (Anell et al., 2012). The included articles discuss aspects of equal health in various contexts and in relation to various age groups, genders, social positions, approaches, competences, and notions of “health religiosity”. The contexts of the studies vary and include elementary schools, mental health-care services and home environments. The cluster comprises research regarding norm criticism in health education, a school-based intervention regarding food and physical activity, clarification of the master’s-level competencies for mental health nursing, peoples’ experiences of living with dysphagia after stroke, and individual health promotion. A life-course approach takes a temporal and societal perspective on the health and well-being of individuals and generations, recognizing that all stages of a person’s life are intricately intertwined with each other as well as with the lives of others born in the same period and with the lives of past and future generations. Adopting a life-course approach involves taking action early in the life-course, taking appropriate action during life’s transitions, and taking action together as a society (WHO, 2018). The five articles to varying degrees focus on a life-course approach. The article on school children’s health behaviours takes a life-course perspective with a positive shift from individual behaviours to peer learning. The workforce development article regarding master’s-level training for mental health nursing competency illustrates the need for a workforce that is aware of the complexity of disadvantage across the life course and the ability to respond to real needs. The article discussing dysphagia following stroke is foremost related to later stages of life but might also refer to health screening in general and its lack of accessible and affordability over the life course. The article on narratives to health beliefs might also fit, because health can be viewed in various ways over the life course. However, the article on norm criticism in health education might provide more of a critical space to challenge normative thinking about tackling health inequalities “in silos” rather than focusing on structural life-course risks and resilience resources. A life-course perspective might be supported by a framework of structural and social determinants of health and well-being. Such social determinants include the circumstances in which people are born, grow, live, work, and age, and they are distributed according to power, wealth, and resources.

Overall, in common for all of the included articles is that they concern equality in people’s health and well-being. All people have equal worth regardless of their gender, age, background, or disability, and persons possessing any kind of a coaching function should treat other human beings with respect (United Nations, 1948). A coaching function might imply an approach that enables learning and development to occur and thus performance to improve in individuals (Parsloe, 1999). Traditionally, society has looked to the health sector to deal with concerns about health and disease. Certainly, the uneven distribution of health care—not delivering care to those who need it the most—is one of the social determinants of health (Mougeot & Naegelen, 2018).

Health inequalities are insufficiently measured, both within countries and globally. The World Health Organization (WHO) (2008) recommends that national governments and international organizations set-up national and global systems for routine monitoring of health inequality and the social determinants of health and for evaluating the health equality impact of different policies and interventions. To create the organizational space and capacity to act effectively on health inequality calls for investment in the training of policy-makers and health practitioners and an understanding of the social determinants of health. In addition, it calls for a stronger emphasis on social determinants in public health research. One social determinant for more equal health is the conditions of daily life—namely the circumstances in which people are born, grow, live, work, and age. The determinant regarding the distribution of power, money, and resources includes the structural influence of conditions of daily life at the global, national, and local levels. Ensuring equal access to health care calls for measurement of the problem, evaluation of action, expansion of knowledge, development of a workforce that is trained in the social determinants of health, and raising public awareness of the social determinants of health (World Health Organization, 2008). One suggested an approach to working with health inequalities described by Pelters (2018a) might be summarized as targeting majorities to support minorities by using norm-criticism in health education. Within norm criticism, the focus is on the norms that limit normative and oppressive structures as well as those that risk repeating such structures (Martinsson & Reimers, 2008). This explains the relevance in cases where there are many standards that are positive and important to preserve and maintain. Norm-critical perspectives are not about dissolving all norms, but about determining which norms we need to counteract and how, and which norms we need to reinforce and confirm (Swedish National Agency for Education, 2016). The article advocates for norm-criticism instead of empowering coping and pedagogy of tolerance as an educational approach for mitigating stigmatization as well as the blame and guilt for health deviant minorities within the field of health disparities. Norm-criticism might be a way of getting members of the (presumably healthy) normative majority to see and to question their health-related norms and to raise awareness of the processes by which members of that majority re-/construct images of stereotypic figures (such as “the fatso” or “the couch potato”) with certain condemnable personal character traits and, in doing so, limit the acting space of those identified as examples of those figures. In conclusion, Pelters (2018a) expressed that norm-criticism is a valuable and much-needed addition to the health intervention repertoire.

The European Commission argues that more action is needed on health-related behaviours such as poor diet and lack of physical activity, and that particular attention should be given to children and young people because poor health, social, or cognitive development at an early age can negatively affect their opportunities later in life. In addition, action on factors affecting health needs to take into account the distribution of health care according to the social group and should aim to ensure that those most in need benefit the most from interventions (European Commission, 2013). The study by Holmberg et al. (2018) aimed to describe adolescents’ experiences of participating in a health-promoting school-based intervention regarding food and physical activity, with a focus on aspects of empowerment. The study was conducted in a school located in a disadvantaged urban community in Sweden characterized by poorer self-reported health and lower life expectancy than the municipality’s average. Focus group interviews with adolescents (n = 49) and their teachers (n = 4) were conducted 2 years after the intervention. The data were categorized using qualitative content analysis, and this generated the theme “Gaining control over one’s health: deciding, trying, and practicing together in new ways using reflective tools” that intersected with all the categories. The adolescents appreciated being able to influence the components of the intervention and having the opportunity to collaborate with peers in active learning activities such as practising sports and preparing meals. They also reported acquiring new health information, that trying new activities was inspiring, and that the use of pedometers and photograph-based food diaries helped them reflect on their health behaviours. The adolescents’ experiences were also echoed by their teachers. To facilitate empowerment and to stimulate learning, health-promotion interventions targeting adolescents might enable active learning activities in groups by using visualization tools to facilitate self-reflection and by allowing adolescents to influence the activities of the intervention. Pelters’ (2018b) paper takes a hermeneutic approach and discusses different meaning horizons in dialogues that focus on the exploration of the variety of narratives that are relevant to people’s health beliefs. Thinking in terms of religiosity as potentially improving health promotion, such as in investigations of the (dis)continuities between the different dimensions of religiosity, might contribute to a deeper understanding and awareness of the complexity of health in general and to the role of morality in particular, as is indicated by healthism.

Previous research has found that people with mental disorders are often denied their right to be treated in an equal way. They are not only discriminated against and marginalized in their communities, but also in the mental health services where they should be receiving care and support (Shekhar & Fahmy, 2015). The WHO Regional Office for Europe (2015) has stipulated that people with mental health problems should foremost be approached in primary care by working in partnership with multidisciplinary mental health staff. However, many people with mental health problems choose not to engage or maintain contact with mental health services due to stigma and discrimination. Discrimination, prejudice, and neglect that hinder people with mental health problems from exercising their rights to equitable access to care must be tackled. They should be approached with accessible, safe, and effective services by competent healthcare staff that meet the mental, physical, and social needs of people with mental health problems. Good mental health service delivery requires competent staff that are able to provide treatments and care that are consistent with the best available evidence. Evidence-based safe and humane interventions and advances in treatment should be reflected in professional curricula and qualifications (WHO Regional Office for Europe, 2015). Jormfeldt et al. (2018) explored the need for a clarified definition of master’s-level mental health nursing competencies in terms of knowledge, skills, and attitudes in a European context. Mental health service users have, in spite of their right to equal overall health, higher rates of physical illness and are more likely to experience premature death than the general population. This implies that it is not enough to only treat the symptoms of mental illness as a measure of the quality of care in mental health services. One main goal of mental health nursing is to provide overall health, but the implementation of a holistic concept of health comprising mental, physical, and social aspects of health in mental health services has previously proven to be challenging. Clarifying master’s-level mental health nursing competencies might contribute to facilitating the promotion of equal overall health care among service users in mental health services. The discussion sheds light on the content, values, and utility of master’s-level mental health nursing competencies in mental health services. Further, the discussion contributes to reduced role ambiguity by distinguishing master’s-level responsibilities from undergraduate nursing tasks and obligations of other professionals in mental health care. This discussion thus shapes the implications for development in master’s-level mental health nursing education curricula.

People with chronic health conditions have been found to be more likely to report difficulties accessing healthcare services. In addition, they have higher odds of experiencing several barriers, including discrimination by healthcare staff, lack of help with communication, lack of information, problems with transportation, and difficulty using facilities (Allerton & Emerson, 2012). A chronic health condition such as stroke is an important cause of disability and mortality in a global view (Lozano et al., 2012; Murray et al., 2012), and previous research has shown that people with low socioeconomic status have increased incidence of stroke (Addo et al., 2012) and increased mortality after stroke (Hanchate, Schwamm, Huang, & Hylek, 2013; Li et al., 2008). However, it is uncertain whether low socioeconomic status is associated with increased functional impairment after stroke. Some previous studies have indicated that low socioeconomic status is associated with functional impairment at 3 months after stroke (Chen et al., 2015; Song et al., 2017). Dysphagia is a common consequence of stroke and is a risk factor for aspiration pneumonia (Martino et al., 2005), which is associated with higher rates of death and disability (Cohen et al., 2016). There is considerable variation in dysphagia screening protocols across geographic areas, and guidelines and performance measures for screening do not specify which protocols are best (Luker, Wall, Bernhardt, Edwards, & Grimmer-Somers, 2010; Smith et al., 2014). Furthermore, it is uncertain whether different swallowing assessments reduce the risk of pneumonia, disability, or death after stroke. Thus, it seems that the care and services for people with dysphagia vary and are unequal. Helldén et al. (2018) investigated peoples’ experiences of living with dysphagia after stroke and their experiences of dysphagia management. Interviews were conducted with five persons with dysphagia after stroke, and these were analysed with qualitative content analysis. The theme “Dysphagia impacts life situations negatively and requires individually adapted, long term support from skilled health care professionals” emerged. The theme included the categories “Learning to manage dysphagia and its complications”, “Professional support with dysphagia varies”, and “Finding small moments of joy despite large restrictions in life situations”. The findings indicated that people with dysphagia often experience a lack of support from healthcare professionals. Actions to increase support might include developing national guidelines for adequate dysphagia follow-up and establishing multidisciplinary dysphagia teams in hospitals and long-term care facilities.

Even though the included articles do not all explicitly express a focus on equality in health, aspects of equality are intrinsically interwoven into all of the groups of people targeted in the papers. In addition, the effects of equality or inequality on the health and well-being of the people involved hopefully will be clear to the reader. We hope that this thematic cluster will supply the reader with a new and broader understanding of equality in health and will deepen their insight into the importance of equality related to people’s health and well-being.
